# Concentric Versus Eccentric Exercise-Induced Fatigue on Proprioception, Motor Control and Performance of the Upper Limb in Handball Players: A Retrospective Study

**DOI:** 10.3390/life16030429

**Published:** 2026-03-06

**Authors:** Stelios Hadjisavvas, Michalis A. Efstathiou, Irene-Chrysovalanto Themistocleous, Manos Stefanakis

**Affiliations:** Department of Health Sciences, University of Nicosia, 2417 Nicosia, Cyprus; efstathiou.m@unic.ac.cy (M.A.E.); themistocleous.i@unic.ac.cy (I.-C.T.); stefanakis.m@unic.ac.cy (M.S.)

**Keywords:** fatigue, proprioception, kinesthesia, sensorimotor control, shoulder, handball, eccentric, concentric, YBT-UQ, ASH test

## Abstract

Background: Upper-limb performance in handball depends on accurate shoulder sensorimotor control under high loads and fatigue. This study examined between-cohort differences associated with concentric versus eccentric exercise-induced fatigue in shoulder proprioception, kinesthesia, functional stability, and isometric force output in professional male handball players. Methods: This was a retrospective, quasi-experimental (non-randomized) between-cohort comparison of two previously collected cohorts who completed either a concentric (*n* = 46) or eccentric (*n* = 33) fatigue protocol, with pre- and post-fatigue assessments of joint repositioning sense (absolute angular error, AAE), threshold to detection of passive movement (TTDPM), Y Balance Test Upper Quarter (YBT-UQ), and the Athletic Shoulder (ASH) test. Results: Fatigue significantly increased AAE across all tested angles (Time: all *p* < 0.001), with a contraction-specific effect at end-range internal rotation (IR45°), where AAE increased more after concentric than eccentric fatigue (Time × Fatigue Type: *p* = 0.017; Δ = +1.34° (+61.8%) vs. +0.20° (+7.4%)). TTDPM increased after fatigue (*p* = 0.001) with no interaction (*p* = 0.968). YBT-UQ performance decreased after fatigue for all dominant-limb outcomes and for non-dominant inferolateral, superolateral, and composite scores (all *p* ≤ 0.018), but not for non-dominant anteromedial reach (*p* = 0.986); no Time × Fatigue Type interactions were detected for YBT-UQ outcomes (all *p* > 0.05). ASH force output decreased across all positions and both limbs (all *p* ≤ 0.002), with the dominant-limb Y position showing a greater decline following eccentric fatigue (Time × Fatigue Type: *p* = 0.030; e.g., ASH Y dominant Δ = −0.49 (−4.6%) vs. −1.43 N·kg^−1^ (−13.3%)). Conclusions: Exercise-induced fatigue impairs shoulder sensorimotor function and upper-limb performance in handball. Contraction-mode differences were small and task-specific in this between-cohort comparison, emerging primarily at end-range proprioception and selected isometric strength positions. These findings may inform the design of training programs that emphasize fatigue-resistant sensorimotor control and end-range strength, while causal inferences regarding contraction mode are not warranted given the non-randomized design.

## 1. Introduction

Handball is a high-intensity, multifaceted sport characterized by frequent overhead actions such as throwing, passing, blocking, and catching, often performed under conditions of accumulated fatigue and high neuromuscular demand. The shoulder complex is exposed to repeated high loads across a large range of motion, and shoulder problems are common in handball populations, particularly when weekly load increases and shoulder strength/control are compromised [1,2].

Muscular fatigue refers to a short-term decline in the capacity to generate force or sustain performance, often accompanied by reduced movement precision and impaired joint stabilization during complex tasks [3]. Fatigue-related performance decline reflects a combination of peripheral changes within the muscle and central alterations in neural drive, both of which can disturb coordinated motor output and joint control [4]. These changes are highly relevant in overhead sports, where the shoulder must maintain dynamic stability while producing and absorbing large torques at high speeds.

The nature of fatigue may also depend on the type of muscle action/contraction used to induce it. Because eccentric actions dominate braking phases, whereas concentric actions dominate acceleration phases, the fatigue produced by these two modes may not be functionally equivalent for the shoulder in overhead sport. Differences in mechanical loading and acute responses could translate to distinct sensorimotor consequences [5]. Understanding whether contraction-specific fatigue differentially affects shoulder sensorimotor function is important for high-performance sport, because sensorimotor deficits could degrade movement consistency and potentially increase vulnerability during demanding phases of throwing [6,7].

Accurate sensing of joint position and movement is critical for coordinating upper-limb actions and maintaining shoulder stability during rapid overhead tasks [8,9]. Prior studies indicate that fatigue can degrade both position sense and movement detection, likely through altered afferent input and less efficient sensorimotor integration [10]. In addition, contraction-specific effects on position sense have been demonstrated experimentally, supporting the plausibility that concentric and eccentric fatigue may not be sensorimotor-equivalent [11]. In the shoulder, muscle fatigue has been shown to compromise proprioception, underscoring the sensitivity of shoulder position sense to fatigue-related changes in muscle mechanoreceptor function [10].

Beyond laboratory measures, fatigue-related sensorimotor impairment may also manifest as reduced functional upper-limb stability. The Upper Quarter Y Balance Test (YBT-UQ) is a reliable closed-chain assessment of upper-extremity reach performance and dynamic stability, making it suitable for detecting fatigue-related changes in functional control [12].

Despite these findings, key gaps remain for translation to overhead sport performance. First, many fatigue–proprioception studies are not conducted in sport-specific samples exposed to repetitive high-velocity throwing demands. Second, prior work often relies on isolated laboratory measures without incorporating functional upper-limb stability outcomes. Third, proprioception is frequently assessed at mid-range positions, whereas end-range shoulder control may be more vulnerable under fatigue and more relevant to late-cocking/transition phases during overhead actions. Therefore, how contraction-specific fatigue influences shoulder sensorimotor function across proprioception, functional stability, and strength in elite handball players remains insufficiently characterized.

Despite the recognized role of fatigue in degrading proprioception and functional control, most research has either focused on the lower limbs or examined fatigue without directly differentiating contraction-specific mechanisms in sport-relevant upper-limb contexts [13]. Therefore, the specific influence of concentric versus eccentric exercise-induced fatigue on shoulder proprioception, sensorimotor function, and performance in handball players remains insufficiently defined.

Accordingly, the purpose of this retrospective study was to compare the effects of concentric versus eccentric exercise-induced fatigue on shoulder proprioception, kinesthesia, dynamic upper-limb stability, and isometric force output in professional handball players. The primary hypothesis was that exercise-induced fatigue would impair proprioception/kinesthesia, functional stability, and isometric force output. A secondary (exploratory) hypothesis was that any differences between eccentric and concentric fatigue would be task-dependent and interpreted cautiously in this retrospective comparison (i.e., not necessarily uniform across all outcomes), with potential divergence most evident in more demanding conditions such as end-range proprioception and selected strength/stability tasks. The null hypothesis was that both fatigue types would produce similar impairments across outcomes. Although prior literature has sometimes suggested greater eccentric-related decrements, in the present retrospective between-cohort comparison any contraction-mode differences were treated as exploratory and interpreted cautiously.

## 2. Methods

### 2.1. Participants

This retrospective comparison study analyzed data from two prior prospective investigations examining the effects of exercise-induced fatigue on shoulder proprioception, sensorimotor function, and performance in male professional handball players. A total of 79 participants were examined: 46 from the concentric exercise study and 33 from the eccentric exercise study. Participants were all right-handed, actively engaged in regular training (minimum three sessions per week) and competitive play at the national level. The sample size of the prospective studies was determined using a priori power analysis in G*Power (3.1.9.7; Heinrich Heine University Düsseldorf, Düsseldorf, Germany), assuming a moderate effect size (0.3), a significance level of 0.05, and a power of 0.82 to detect differences between pre- and post-fatigue measures. Because participants were not randomly allocated to contraction mode and the analysis compares two independently collected cohorts, the present study constitutes a retrospective, quasi-experimental (non-randomized) between-cohort comparison. Therefore, findings should be interpreted as associations between contraction mode and post-fatigue outcomes rather than causal effects attributable solely to contraction mode.

Participant characteristics are presented in Table 1. Groups were comparable for age, height, weight, BMI, and playing position distribution (all *p* > 0.05). All participants provided informed consent for their data to be used in this retrospective analysis. Ethical approval for the original data collection was granted by the respective institutional committees. Because the study compares two independently collected cohorts, detailed information regarding training phase and competitive load at the time of testing (e.g., microcycle context, match congestion) was not consistently available; therefore, residual contextual differences and unmeasured confounding cannot be fully excluded, further limiting causal interpretation.

### 2.2. Inclusion and Exclusion Criteria

Participants were included if they were male professional handball players aged 18–35 years, with no history of shoulder injury or surgery within the previous six months, and without neurological conditions or congenital shoulder stiffness. Exclusion criteria consisted of the presence of any shoulder pain, discomfort during initial clinical tests, or a positive orthopedic special test during screening (such as Neer’s, Hawkins-Kennedy, or Yergason’s tests). Additionally, any participant reporting more than two abnormal clinical findings was excluded from the original data set. For this retrospective comparison, only complete datasets with valid pre- and post-fatigue assessments were analyzed.

### 2.3. Experimental Protocol

This retrospective, between-cohort comparison is best conceptualized as quasi-experimental (non-randomized), as contraction mode was not randomized and participants were not assessed within a unified within-subject (crossover) design; accordingly, causal effects of contraction mode cannot be inferred. The included data originated from two prior studies employing a pre-test and post-test design. In both cases, the same assessment protocols for proprioception, sensorimotor function, and performance were used before and immediately after a fatigue-inducing exercise protocol. Participants first underwent anthropometric measurements, including height and weight, measured with standardized electronic tools. They then completed shoulder screening, including active and passive range of motion, manual isometric muscle testing, and orthopedic special tests to confirm shoulder health.

**Figure 1:**
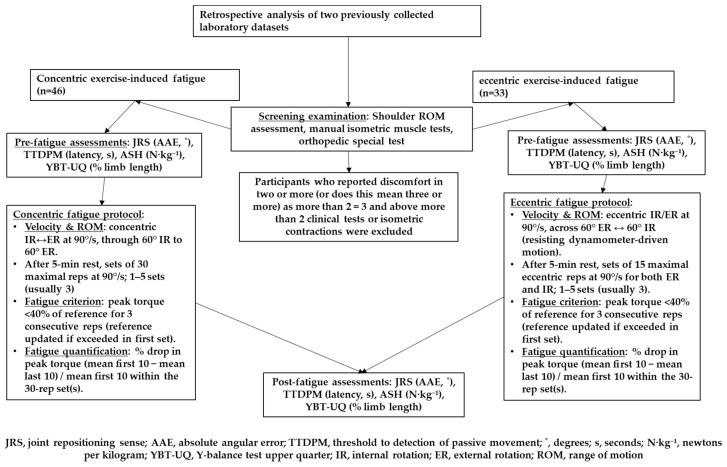
Retrospective analysis of two previously collected laboratory datasets and testing sequence. Screening examination and eligibility, pre-fatigue assessments (JRS—AAE, TTDPM—latency, ASH—normalized force, YBT-UQ), contraction-specific fatigue protocol (concentric vs. eccentric), and immediate post-fatigue reassessments.

Following screening, the assessments included joint repositioning sense (JRS), threshold to detection of passive movement (TTDPM), Athletic Shoulder (ASH) test, and Y balance test–Upper Quarter (YBT-UQ). All assessments were conducted under controlled conditions, with participants wearing minimal attire and blinded to the position of their limbs during proprioception tests to prevent visual cues. An overview of the retrospective study design and testing sequence for both datasets is provided in Figure 1. The two datasets were collected during the same season period and within the same laboratory setting. All assessments were administered by the same examiners using the same equipment, standardized instructions, and identical pre–post testing order (Figure 1). Testing conditions (laboratory environment, participant positioning/strapping, and visual occlusion procedures for proprioception tests) were kept consistent across datasets to maximize comparability. However, because the datasets were collected independently, complete documentation of the competitive/training context at the time of testing was not uniformly available for all participants.

### 2.4. Measurement of JRS

JRS was assessed using an isokinetic dynamometer in a standing position. Participants’ shoulders were abducted to 30° in the scapular plane (30° anterior to the frontal plane), with the elbow flexed to 90° in a neutral position. The arm was secured in the dynamometer to ensure consistent movement. This testing position and setup are commonly used in shoulder proprioception research to assess joint position sense under controlled conditions [8,10]. Before testing, participants performed warm-up repetitions—specifically, two sets of 15 shoulder internal and external rotation exercises at 90°/s—to familiarize themselves with the test movements.

For the assessment, six target angles were used: 15°, 30°, and 45° of internal rotation; and 15°, 30°, and 45° of external rotation. These angles were selected based on previous studies investigating shoulder proprioception across functional ranges of motion [9,10]. The specific target angles were the same for all participants. The examiner passively positioned the shoulder at each target angle, maintained that position for 3 s to enhance kinesthetic memory, and then returned the shoulder to the starting position. The participant then actively attempted to reproduce the target angle and hold it for 3 s. The examiner recorded the response, and the process was repeated three times per angle. The average of the three trials was used to calculate the absolute angular error (AAE) as the absolute difference between the target and the reproduced angle, as recommended in proprioception research [8]. The order of target angles and instructions were identical pre- and post-fatigue, and standardized rest intervals were provided between trials. Dynamometer calibration (and gravity correction if applicable) followed manufacturer guidelines.

### 2.5. Measurement of TTDPM

TTDPM was also assessed with the participant in the same position on the dynamometer as in the JRS test. Once in position, visual and auditory cues were minimized using a soft eye mask and earplugs. From 45° external rotation, the dynamometer passively rotated the shoulder into internal rotation at 0.5°/s. Participants pressed a stop button at the instant they perceived movement onset, and the time delay between movement onset and the stop action was recorded. The primary TTDPM outcome was detection latency (s), defined as the time elapsed from the onset of dynamometer movement to the participant’s button press (mean of three trials). The mean time of three trials was used in the analysis [9,10].

### 2.6. Measurement of YBT-UQ

Participants started in a push-up position with feet shoulder-width apart. Using the YBT kit™, they moved the indicator plate by pushing with their hand as far as possible in three specified directions: anteromedial (AM), superolateral (SL), and inferolateral (IL). Prior to testing, a warm-up of three attempts in each direction was performed. Subsequently, three additional attempts per direction were conducted, with the maximum reach distance recorded for each trial. The scores in each direction were summed to generate a composite reach distance, which was then normalized to upper limb length (measured from the seventh cervical vertebra to the tip of the middle finger). A failure was recorded if the participant lost control, contacted the floor with their hand, or had their feet lose contact with the ground. High intra-test reliability (ICC 0.80–1.0) in previous studies supports its validity for assessing dynamic shoulder stability and neuromuscular control [12,14].

### 2.7. Measurement of ASH Test

The ASH test assessed shoulder isometric strength in three positions: “I” (full abduction), “Y” (135° abduction), and “T” (90° abduction). Participants lay prone with their foreheads resting on a cushion. The hand was placed on a force plate with the palm facing downward. For each position, the participant exerted maximum force against the force plate for 3 s while the examiner recorded the peak force at 300 Hz. Peak force was normalized to body mass and reported as N·kg^−1^ (peak force in N divided by body mass in kg). Three trials per position were performed with rest intervals, and the best effort was used for analysis. The ASH test values demonstrated excellent inter-day reliability (ICC 0.94–0.98) and are indicative of shoulder strength relevant to handball performance [15].

### 2.8. Fatigue Protocol (Concentric Versus Eccentric)

The concentric exercise-induced fatigue protocol involved participants completing a warm-up consisting of two sets of 15 submaximal concentric shoulder rotations at 90°/s to familiarize themselves with the movement pattern and prepare the muscles. Following the warm-up, they performed 10 maximal concentric repetitions at 90°/s for both internal and external shoulder rotation, with the highest peak torque serving as the baseline or reference value. After a 5-min rest period, participants performed multiple sets of 30 maximal-effort concentric contractions for both internal and external rotator muscles. These repetitions involved pushing from 60° of internal rotation to 60° of external rotation repeatedly at 90°/s, with instructions to exert maximum effort throughout. The fatigue criterion was set such that fatigue was considered achieved when the peak torque dropped below 40% of the baseline value and remained below this threshold across three consecutive repetitions within a set [3]. If a participant’s peak torque exceeded the initially recorded baseline in any subsequent set, the reference value used for the <40% criterion was updated to the highest peak torque observed (rolling maximum), and the set continued. This procedure was implemented to account for occasional post-warm-up potentiation/learning effects, ensuring that the reference value reflected the participant’s highest observed maximal capacity rather than an underestimated initial effort. Because baseline updating can only increase the reference value, it renders the fatigue criterion more conservative (i.e., harder to reach <40% of baseline), and it was applied consistently across participants and in both protocols. The sets were performed until this criterion was met, which typically occurred after three to five sets per muscle group. The percentage decrease in peak torque between baseline (i.e., the highest observed peak torque) and the post-fatigue effort was used to verify attainment of the predefined fatigue criterion. To characterize the within-task torque-decline pattern, we additionally quantified the percentage drop from the first 10 to the last 10 repetitions (Table S1), which provides a complementary fatigue index that is not dependent on the baseline-updating rule.

The eccentric fatigue protocol was designed to induce fatigue through resistance during lengthening contractions. Similarly, participants performed two warm-up sets of 15 submaximal eccentric shoulder rotations at 90°/s from 60° external to 60° internal rotation to familiarize themselves with eccentric movement patterns. The baseline peak torque was then measured during 10 maximal-effort eccentric repetitions at 90°/s, with the highest torque value recorded. After a five-minute rest, participants performed sets of 15 maximal eccentric contractions in both directions—resisting movement from 60° external to 60° internal rotation—at the same speed. The fatigue criterion for the eccentric protocol was identical to that for the concentric protocol: fatigue was considered achieved when the peak torque dropped below 40% of the baseline value for three consecutive repetitions. Although the torque-based criterion confirms substantial performance decline, it does not guarantee equivalence of underlying neuromuscular or sensorimotor fatigue between contraction modes; thus, workload/fatigue equivalence should be interpreted as approximate within the limits of retrospective data. During the effort, the examiner ensured proper resistance and controlled movement, and the reference value used for the <40% criterion was updated to the highest peak torque observed (rolling maximum) if efforts exceeded the initial value in any subsequent set. The eccentric sets continued until the criterion was met, and the percentage decrease in peak torque was calculated using the same formula as the concentric protocol. These contraction-specific fatigue protocols align with previous research examining neuromuscular and sensorimotor responses to concentric and eccentric loading [5]. To characterize the torque-decline pattern, the concentric dataset was quantified as the percentage drop in peak torque from the first 10 to the last 10 repetitions: (mean first 10 − mean last 10)/mean first 10 × 100. For the eccentric dataset, fatigue confirmation was summarized as the percentage change in peak torque from pre- to post-fatigue: (Before − After)/Before × 100 (Table S1). Total sets/repetitions completed until the fatigue criterion and time-to-fatigue were not consistently available in the retrospective records; therefore, protocol comparability in workload was documented primarily via the predefined fatigue criterion and the torque-decline metrics reported in Table S1. Perceptual and physiological markers of fatigue (e.g., RPE, EMG, or metabolic measures) were not collected the retrospective datasets, limiting mechanistic comparison of fatigue between contraction modes.

### 2.9. Statistical Analysis

Statistical analyses were performed using Jamovi software (Version 2.3.26). Data were initially screened for normality and homogeneity of variance using the Shapiro–Wilk and Levene’s tests, respectively, and descriptive statistics were calculated for all variables (mean ± standard deviation). To examine the effects of exercise-induced fatigue type (concentric vs. eccentric) on proprioception, sensorimotor function, and upper-limb performance, a series of mixed-design analyses of variance (2 × 2) were conducted with Time (pre-fatigue, post-fatigue) as the within-subjects factor and Fatigue Type (concentric, eccentric) as the between-subjects factor. Separate mixed ANOVAs were performed for absolute angular error (AAE) across six shoulder joint angles (ER15°, ER30°, ER45°, IR15°, IR30°, IR45°), threshold to detection of passive movement (TTDPM) for the shoulder internal rotation, Y Balance Test–Upper Quarter (YBT-UQ) outcomes across limb (dominant, non-dominant) and direction (anteromedial, superolateral, inferolateral), and Athletic Shoulder (ASH) test outcomes across limb (dominant, non-dominant) and arm position (I, Y, T). For each analysis, main effects of Time and Fatigue Type, as well as their interaction (Time × Fatigue Type), were examined to determine whether pre-to-post changes differed between fatigue protocols. Effect sizes were reported as partial eta squared (η^2^p), the level of statistical significance was set at *p* < 0.05, and Greenhouse–Geisser corrections were applied when the assumption of sphericity was violated. The magnitude of effect sizes was deemed as: small η^2^p ≤ 0.01, medium η^2^p ≥ 0.06, and large η^2^p ≥ 0.14. All graphical representations and estimated marginal means were generated within Jamovi to visualize Time × Fatigue Type effects for each outcome measure.

Given the number of outcomes tested across domains (JRS angles, YBT-UQ directions/limbs, and ASH positions/limbs), we addressed multiplicity by applying a false discovery rate (FDR) adjustment (Benjamini–Hochberg) within each outcome family for the primary effects of interest (Time and Time × Fatigue Type). Findings that did not remain significant after adjustment were interpreted as exploratory. In addition to η^2^p, we reported mean pre–post changes within each fatigue cohort with 95% confidence intervals (and, where appropriate, standardized within-group effects) to facilitate interpretation of practical magnitude beyond *p*-values. Full mixed-design ANOVA outputs re-computed from the shared dataset are provided in Appendix A (Table A1, Table A2 and Table A3).

## 3. Results

Because multiple outcomes were tested, we applied a false discovery rate (Benjamini–Hochberg) adjustment within each outcome family (JRS, YBT-UQ, ASH) for the primary effects of interest (Time and Time × Fatigue Type). Findings that did not remain significant after adjustment should be interpreted as exploratory, and emphasis is placed on consistent patterns across outcomes rather than isolated effects. Accordingly, the Results narrative prioritizes the overall fatigue (Time) effects, while Time × Fatigue Type interactions are reported as secondary, task-specific findings. Mean pre–post changes with 95% confidence intervals are reported for key outcomes to support interpretation of practical magnitude alongside *p*-values.

### 3.1. Fatigue Confirmation

In the concentric protocol, peak torque declined substantially within the task, with mean reductions of 62.05% for IR and 60.00% for ER from the first to the last 10 repetitions (Table S1). In the eccentric protocol, peak torque decreased from pre- to post-fatigue by 65.79% (IR) and 67.93% (ER) (Table S1), confirming that both protocols induced marked fatigue. Importantly, the fatigue-confirmation indices reported in Table S1 (concentric: first-10 vs. last-10 repetitions; eccentric: pre-to-post change) are independent of the baseline-updating procedure, supporting that the documented torque reductions are robust to this standardization rule.

### 3.2. Joint Repositioning Sense (JRS)

Mixed-design ANOVA revealed significant fatigue-related increases in AAE at all tested shoulder rotation angles (Time: all *p* < 0.001; Table 2), indicating reduced joint repositioning accuracy after both protocols. Mean pre–post increases in AAE were observed across angles for both cohorts (Concentric: ER15° Δ = +2.81° [95% CI: 2.12 to 3.50], ER30° Δ = +2.71° [1.98 to 3.44], ER45° Δ = +3.22° [2.19 to 4.25], IR15° Δ = +1.15° [0.71 to 1.58], IR30° Δ = +2.09° [1.43 to 2.75], IR45° Δ = +1.34° [0.69 to 1.99]; Eccentric: ER15° Δ = +2.14° [1.52 to 2.76], ER30° Δ = +2.64° [1.89 to 3.38], ER45° Δ = +2.21° [1.44 to 2.98], IR15° Δ = +1.18° [0.50 to 1.86], IR30° Δ = +1.06° [0.20 to 1.92], IR45° Δ = +0.20° [−0.45 to 0.86]). Across angles, the dominant pattern was a generalized fatigue-related deterioration (Time effects), whereas contraction-mode differences were limited. A significant Time × Fatigue Type interaction was observed only at IR45° (F(1,77) = 5.91, *p* = 0.017, η^2^p = 0.07; Table 2), where AAE increased more following concentric fatigue (Δ = +1.34° [0.69 to 1.99]) than eccentric fatigue (Δ = +0.20° [−0.45 to 0.86]). No interaction effects were detected at the remaining angles (all *p* > 0.05).

### 3.3. Kinesthesia–Threshold to Detection of Passive Movement (TTDPM)

TTDPM increased significantly following fatigue (Time: F(1,77) = 10.86, *p* = 0.001, η^2^p = 0.12; Table 3), reflecting reduced kinesthetic sensitivity. No Time × Fatigue Type interaction was observed (F(1,77) = 0.00, *p* = 0.968), indicating comparable fatigue-related effects between protocols. The mean change in TTDPM was Δ = +1.48 s (95% CI: 0.17 to 2.79) after concentric fatigue and Δ = +1.52 s (95% CI: 0.30 to 2.73) after eccentric fatigue.

### 3.4. Dynamic Upper-Limb Stability–Y Balance Test Upper Quarter (YBT-UQ)

YBT-UQ reach performance declined after fatigue in the dominant limb for anteromedial, inferolateral, superolateral, and composite scores (Time: all *p* ≤ 0.003; Table 4). In the dominant limb, mean changes were: anteromedial Δ = −1.76 (95% CI: −3.86 to 0.34) after concentric fatigue and Δ = −2.85 (95% CI: −4.79 to −0.90) after eccentric fatigue; inferolateral Δ = −2.48 (95% CI: −5.20 to 0.24) vs. Δ = −5.97 (95% CI: −8.88 to −3.06); superolateral Δ = −2.93 (95% CI: −4.93 to −0.94) vs. Δ = −4.12 (95% CI: −6.00 to −2.25); and composite Δ = −2.56 (95% CI: −4.44 to −0.68) vs. Δ = −4.65 (95% CI: −6.80 to −2.50), for concentric and eccentric protocols respectively.

In the non-dominant limb, fatigue-related reductions were evident for inferolateral, superolateral, and composite scores (Time: all *p* ≤ 0.018), with mean changes of inferolateral Δ = −2.74 (95% CI: −5.02 to −0.46) vs. Δ = −3.12 (95% CI: −6.35 to 0.11); superolateral Δ = −1.43 (95% CI: −3.23 to 0.36) vs. Δ = −2.27 (95% CI: −4.65 to 0.10); and composite Δ = −1.28 (95% CI: −2.87 to 0.30) vs. Δ = −2.33 (95% CI: −5.00 to 0.33), for concentric and eccentric protocols respectively. Non-dominant anteromedial reach did not change (F(1,77) = 0.00, *p* = 0.986), with mean changes of Δ = +0.59 (95% CI: −1.21 to 2.39) after concentric fatigue and Δ = −0.85 (95% CI: −3.22 to 1.52) after eccentric fatigue. A main effect of Fatigue Type was detected only for dominant anteromedial reach (F(1,77) = 4.26, *p* = 0.042, η^2^p = 0.05; Table 4). Because the Time × Fatigue Type interaction for dominant anteromedial reach was not significant, this between-group main effect likely reflects baseline differences between cohorts rather than a differential fatigue response, and should be interpreted accordingly. No Time × Fatigue Type interactions were detected for all other YBT-UQ outcomes (all *p* > 0.05; Table 4).

### 3.5. Athletic Shoulder (ASH) Test

Isometric shoulder force output decreased significantly following fatigue across all ASH positions and both limbs (Time: all *p* ≤ 0.002; Table 5). Mean pre–post changes (N·kg^−1^) were: Dominant I Δ = −1.16 (95% CI: −2.02 to −0.29) after concentric fatigue and Δ = −1.37 (95% CI: −2.38 to −0.36) after eccentric fatigue; Dominant Y Δ = −0.50 (95% CI: −1.10 to 0.11) vs. Δ = −1.43 (95% CI: −1.98 to −0.88); Dominant T Δ = −0.37 (95% CI: −0.83 to 0.08) vs. Δ = −1.02 (95% CI: −1.44 to −0.59). In the non-dominant limb, changes were: I Δ = −1.43 (95% CI: −1.93 to −0.93) vs. Δ = −0.87 (95% CI: −1.41 to −0.33); Y Δ = −0.77 (95% CI: −1.24 to −0.30) vs. Δ = −0.82 (95% CI: −1.58 to −0.05); and T Δ = −0.74 (95% CI: −1.23 to −0.24) vs. Δ = −0.46 (95% CI: −1.10 to 0.18), for concentric and eccentric protocols respectively.

Contraction-mode effects were task-specific: Time × Fatigue Type interactions were observed for the dominant-limb Y and T positions (Y: F(1,77) = 4.90, *p* = 0.030, η^2^p = 0.06; T: F(1,77) = 4.01, *p* = 0.049, η^2^p = 0.05; Table 5), with larger post-fatigue reductions after the eccentric protocol. However, the ASH dominant T interaction (*p* = 0.049) did not remain significant after within-family FDR adjustment (q≈0.061) and should therefore be interpreted as exploratory. The dominant-limb Y interaction remained significant after within-family FDR adjustment and is interpreted as a task-specific contraction-mode difference. No interaction effects were detected for the remaining ASH outcomes (all *p* > 0.05).

## 4. Discussion

The purpose of this retrospective study was to compare the effects of concentric versus eccentric exercise-induced fatigue on shoulder proprioception, kinesthetic sensitivity, functional upper-limb stability, and isometric strength in professional handball players. The most robust finding was a consistent time effect of fatigue, producing broad impairments across sensory (JRS, TTDPM) and functional (YBT-UQ, ASH) domains. Importantly, the majority of observed impairments were explained by fatigue itself (Time effects), whereas contraction-mode differences were limited and emerged only under restricted task and joint-position conditions. In contrast, contraction-mode differences should be interpreted more cautiously, as they were neither frequent nor global; instead, they were task- and position-specific, emerging only at end-range internal rotation for joint repositioning accuracy and in selected ASH positions of the dominant limb. Given the retrospective comparison of two pre-existing cohorts and the inherent differences in protocol structure/available confirmation metrics, contraction-mode effects may reflect a combination of fatigue physiology and cohort/protocol factors rather than a uniform “superiority” of one contraction mode. Because this is a retrospective comparison of two independent, previously collected cohorts rather than a randomized or within-subject design, between-protocol inferences should be interpreted cautiously. These interaction effects were limited in number and of small-to-moderate magnitude; therefore, they should be considered preliminary signals requiring confirmation in prospective within-subject studies. The primary hypothesis was supported, as fatigue (Time effects) impaired outcomes across domains. Secondary hypotheses regarding contraction-mode differences were only partially supported, with interactions limited to IR45° JRS and the dominant-limb ASH Y position, while most outcomes showed comparable responses between cohorts; the analogous ASH T finding should be interpreted as exploratory after multiplicity control. Notably, our initial directional expectation of greater eccentric-related deficits was not supported for proprioception, as the only clear interaction (IR45°) showed a larger deterioration following concentric fatigue, underscoring the task- and joint-position–specific nature of contraction-mode effects. Mechanistic interpretations should be considered hypothesis-generating, as the study did not include EMG, measures of central fatigue, kinematic analyses, or neuromuscular activation profiling.

### 4.1. Fatigue-Related Impairment of Shoulder Proprioception

A clear deterioration in joint position sense was observed following fatigue across all tested shoulder rotation angles, with moderate-to-large effect sizes. One possible explanation is that fatigue may alter afferent signaling (e.g., spindle sensitivity) and its central integration, which could reduce the precision of joint angle reproduction [8,9]. In handball, where repetitive overhead throwing requires precise shoulder positioning under high loads, such proprioceptive degradation may compromise movement accuracy and joint stability.

Importantly, a Time × Fatigue Type interaction was detected at 45° of internal rotation, indicating contraction-specific modulation of proprioceptive accuracy at an end-range joint position. Notably, the increase in repositioning error was larger after concentric fatigue than after eccentric fatigue, suggesting that contraction mode may influence end-range proprioceptive processing when the shoulder operates near mechanical constraints. One potential explanation is that near end-range IR, repositioning accuracy may rely more heavily on active force modulation and afferent feedback from fatigued internal rotators; thus, concentric fatigue of the agonist musculature may produce a larger error when joint constraints and sensorimotor demands are highest. These mechanistic interpretations remain speculative because no direct neurophysiological measures were collected. This joint-position specificity is practically relevant to throwing, as end-range control is challenged during late cocking and deceleration phases when rapid sensorimotor adjustments are required [6,7]. Importantly, this finding differs from our initial expectation that eccentric fatigue would uniformly induce greater deficits, supporting a more nuanced, task- and position-dependent interpretation of contraction-mode effects. Thus, our initial directional expectation of greater eccentric-related proprioceptive impairment was not supported for the end-range JRS outcome (IR45°), reinforcing that contraction-mode effects—when present—are likely task- and joint-position dependent.

The absence of interaction effects at mid-range angles suggests that fatigue-related proprioceptive impairments are largely generalized across contraction modes, except when joint demands approach extremes of range or load. This supports the notion that contraction-specific fatigue effects may become more evident under conditions of heightened sensorimotor uncertainty or mechanical constraint. Because minimal detectable change (MDC) values are not consistently established for this exact dynamometer-based JRS protocol, the practical relevance of these changes is best interpreted using the observed mean changes with confidence intervals and the consistency of time effects across angles.

### 4.2. Kinesthetic Sensitivity and Afferent Processing Under Fatigue

Fatigue also resulted in significant reductions in kinesthetic sensitivity, as reflected in increased thresholds for detecting passive movement. The rise in TTDPM after fatigue may indicate reduced sensitivity to small passive movements, consistent with reports of fatigue-related changes in movement-related sensory processing [9,16]. The lack of a contraction-type interaction suggests that both concentric and eccentric fatigue similarly disrupt afferent signal fidelity at low movement velocities [4,9,16,17,18,19].

From a motor control perspective, impaired kinesthetic feedback may reduce the nervous system’s ability to detect subtle perturbations or movement onset, potentially delaying corrective responses during dynamic tasks. In overhead sports such as handball, where shoulder stability relies heavily on rapid sensorimotor integration, such deficits may increase reliance on feedforward control strategies, which are less adaptable under unpredictable conditions [4]. These mechanistic interpretations should be considered hypothesis-generating, as no direct neurophysiological measures were collected. However, minimal detectable change values are not consistently established for this exact dynamometer-based TTDPM protocol; therefore, practical interpretation is supported by the observed mean changes with confidence intervals and the consistency of fatigue effects across cohorts.

### 4.3. Functional Upper-Limb Stability and Dynamic Motor Control

The observed reductions in YBT-UQ performance following fatigue are consistent with impaired sensorimotor control at the functional level. Significant main effects of time across all dominant-limb reach directions and composite scores indicate a generalized decline in dynamic upper-limb stability. Similar fatigue-related decrements in YBT-UQ performance have also been reported in adolescent handball players, alongside reductions in throwing performance . Declines in YBT-UQ performance suggest that that fatigue-related sensory changes may translate to measurable reductions in closed-chain stability and reach control [14,20].

Although a main effect of Fatigue Type was observed for dominant anteromedial reach (Table 4), no further Time × Fatigue Type interactions were detected. Because the Time × Fatigue Type interaction was not significant, this main effect likely reflects baseline cohort differences rather than different fatigue responses between protocols. This indicates that the magnitude of the fatigue-induced decline in dynamic stability was comparable between protocols. From a practical standpoint, previously reported YBT-UQ minimal detectable change thresholds (~6–8 cm depending on direction, and ~6.5–6.9 for the composite score) provide a useful anchor for judging whether post-fatigue reductions are likely to exceed typical measurement error [12].

### 4.4. Isometric Force Production and Neuromuscular Capacity

The ASH test revealed consistent reductions in isometric shoulder force output across all positions and both limbs following fatigue, with moderate-to-large effect sizes. These results are consistent with fatigue-related reductions in force-generating capacity and align with previous research demonstrating fatigue-related declines in maximal voluntary force output following both concentric and eccentric loading [3].

While fatigue reduced isometric force output across all ASH positions, contraction-mode effects were limited and task-specific. In the dominant limb, the Y position showed a greater post-fatigue strength loss following eccentric loading, and this interaction remained significant after within-family FDR adjustment. In contrast, the corresponding effect in the T position was borderline (*p* = 0.049) and did not remain significant after FDR correction, and should therefore be interpreted as exploratory. These position-specific patterns may reflect task-dependent motor unit recruitment strategies and/or greater peripheral fatigue during eccentric work in shoulder abduction positions relevant to handball and similar overhead populations; however, mechanistic interpretation remains hypothesis-generating because no direct neuromuscular or neurophysiological measures (e.g., EMG) were collected. For context, published ASH reliability work has reported minimum detectable change values on the order of ~13–26 N (position-dependent). Although our ASH outcomes were normalized (N·kg^−1^), these thresholds provide a practical anchor for interpreting whether post-fatigue reductions are likely to represent meaningful decrements rather than measurement noise [15].

### 4.5. Implications for Sensorimotor Control in Handball

Taken together, the findings indicate that exercise-induced fatigue compromises shoulder sensorimotor function at multiple levels in asymptomatic, right-handed male professional handball players, including proprioceptive accuracy, kinesthetic sensitivity, dynamic stability, and force production. Importantly, fatigue itself (Time effects) explained the majority of observed impairments across outcomes, whereas contraction-specific effects were limited and emerged only under restricted task and joint-position conditions. While contraction-specific effects were limited, the interaction observed at end-range internal rotation highlights the importance of considering joint position and task demands when evaluating fatigue-related neuromuscular deficits. Accordingly, the strongest practical implication is the broad fatigue-related decline across domains, whereas contraction-mode differences should be viewed as secondary and outcome-specific.

In practice, the results support monitoring and training approaches that prioritize the development of fatigue-resistant shoulder sensorimotor control across proprioception, functional stability, and strength. Assessments may be particularly informative when performed post-fatigue and near end-range positions relevant to overhead actions. Training may combine end-range repositioning/perturbation drills with progressive closed-chain stability tasks and isometric strengthening across multiple shoulder positions to improve tolerance to fatigue. Contraction-mode–specific implications should be viewed as preliminary in this retrospective cohort comparison; therefore, these findings should not be interpreted as indicating a preferential practical role for concentric versus eccentric loading. Instead, both contraction modes can be incorporated within comprehensive shoulder programs, and prospective trials are needed to determine whether tailoring training based on contraction mode yields meaningful performance or injury-related benefits [21,22]. In addition, throwing-specific biomechanics across phases such as late cocking and deceleration should be considered when interpreting end-range shoulder demands and designing sport-relevant interventions [23].

### 4.6. Limitations and Future Directions

Several limitations should be acknowledged. The central limitation of this study is the retrospective integration of two independently collected cohorts rather than a randomized or within-subject crossover design. As with any retrospective cohort comparison, selection bias cannot be excluded, and unmeasured differences between cohorts may have influenced between-group and interaction effects. This structural constraint limits causal inference regarding contraction mode and prevents full control of cohort-level contextual factors (e.g., training/competitive load), allowing residual confounding despite identical laboratory procedures and standardized testing conditions. Consequently, robust causal inferences regarding contraction mode cannot be made, and contraction mode cannot be assumed to be the only systematic difference between cohorts. Specifically, variables such as training microcycle phase, weekly training/competitive load, and history of shoulder overuse, were not uniformly documented across the original studies; therefore, these potential confounders cannot be fully ruled out when interpreting between-cohort (Fatigue Type) and interaction effects. Second, the torque-based fatigue criterion confirms substantial performance decline but does not guarantee equivalence of underlying neuromuscular or sensorimotor fatigue between concentric and eccentric protocols; therefore, physiological equivalence between modes cannot be assumed. Third, mechanistic interpretations are necessarily limited because no direct neuromuscular or neurophysiological measurements (e.g., EMG, metabolic or perceptual markers) were available to characterize fatigue mechanisms and afferent processing; therefore, mechanistic explanations should be considered hypothesis-generating. Fourth, only male professional handball players were included, limiting generalizability to other populations and levels of play. Moreover, the large number of outcomes and statistical tests increases the risk of type I error; thus, isolated significant findings—particularly interaction effects—should be interpreted cautiously and confirmed in prospective studies.

Future research should explore the interaction between fatigue type, joint position, and task complexity, as well as the long-term adaptations to concentric and eccentric loading on sensorimotor control. Future trials should prioritize within-subject crossover designs with matched workload and time-to-fatigue and include neuromuscular/neurophysiological measures (e.g., EMG) to isolate contraction-mode effects and test mechanistic hypotheses more cleanly. Such work would further clarify how fatigue mechanisms influence movement coordination in high-performance overhead sports.

## 5. Conclusions

Fatigue substantially impaired proprioception, kinesthetic sensitivity, functional upper-limb stability, and ASH isometric force output, indicating a robust overall negative impact of neuromuscular fatigue on shoulder sensorimotor function and upper-limb performance in professional handball players. Contraction-mode differences were limited and outcome-specific and should be considered preliminary in this retrospective cohort comparison: concentric fatigue was associated with a larger increase in repositioning error at end-range internal rotation, whereas eccentric fatigue was associated with a greater reduction in dominant-limb ASH force output in the Y position, with the analogous finding in the T position interpreted as exploratory after multiplicity control. Collectively, these findings support a rationale for emphasizing fatigue-resistant sensorimotor and strength training, particularly targeting end-range shoulder control and abduction positions relevant to throwing. Future research should employ a within-subject crossover design with matched workload and time-to-fatigue to isolate contraction-mode effects more cleanly and should test whether targeted training interventions translate into meaningful performance benefits and reduced injury risk in prospective trials.

These conclusions are most directly applicable to male, professional, right-handed handball players without shoulder symptoms, and should be generalized to other overhead athletes (e.g., females, youth, symptomatic players, left-handed athletes, or different competitive levels) with caution.

## Figures and Tables

**Table 1:** Participant characteristics by fatigue protocol (mean ± SD or *n* (%)).

Variable	Concentric (*n* = 46)	Eccentric (*n* = 33)	*p*-Value
Age (years)	26.1 ± 5.5	25.7 ± 5.4	0.786
Height (cm)	179.9 ± 5.5	179.1 ± 5.3	0.522
Weight (kg)	84.9 ± 13.3	83.5 ± 13.0	0.644
BMI (kg/m^2^)	26.2 ± 3.9	26.0 ± 4.0	0.837
Playing position, *n* (%)			0.460
Offensive	20 (43.5%)	19 (57.6%)	
Defensive	21 (45.7%)	11 (33.3%)	
Goalkeeper	5 (10.9%)	3 (9.1%)	

Abbreviations: BMI, body mass index. Notes: Values are mean ± SD unless otherwise indicated. *p*-values are from Welch’s independent *t*-tests (continuous variables) and a chi-square test (position).

**Table 2:** Joint repositioning sense (JRS): absolute angular error (AAE) across rotation angles (mean ± SD) and key ANOVA effects.

Outcome	Concentric Pre	Concentric Post	Eccentric Pre	Eccentric Post	Time	Time × Fatigue Type
ER15° AAE	1.91 ± 0.96	4.72 ± 1.98	1.95 ± 0.98	4.09 ± 1.80	F(1,77) = 114.49, *p* < 0.001, η^2^p = 0.60	F(1,77) = 1.95, *p* = 0.166, η^2^p = 0.02
ER30° AAE	2.64 ± 1.21	5.35 ± 2.49	2.78 ± 1.38	5.41 ± 2.26	F(1,77) = 105.37, *p* < 0.001, η^2^p = 0.58	F(1,77) = 0.02, *p* = 0.890, η^2^p = 0.00
ER45° AAE	2.69 ± 1.36	5.91 ± 3.33	2.53 ± 1.14	4.74 ± 2.14	F(1,77) = 68.74, *p* < 0.001, η^2^p = 0.47	F(1,77) = 2.16, *p* = 0.146, η^2^p = 0.03
IR15° AAE	1.97 ± 1.01	3.11 ± 1.48	2.22 ± 1.02	3.40 ± 1.72	F(1,77) = 38.01, *p* < 0.001, η^2^p = 0.33	F(1,77) = 0.01, *p* = 0.924, η^2^p = 0.00
IR30° AAE	2.60 ± 1.25	4.69 ± 2.43	3.08 ± 1.10	4.14 ± 2.36	F(1,77) = 40.72, *p* < 0.001, η^2^p = 0.35	F(1,77) = 3.79, *p* = 0.055, η^2^p = 0.05
IR45° AAE	2.17 ± 0.90	3.51 ± 2.03	2.69 ± 1.48	2.89 ± 1.75	F(1,77) = 14.03, *p* < 0.001, η^2^p = 0.15	F(1,77) = 5.91, *p* = 0.017, η^2^p = 0.07

Abbreviations: AAE, absolute angular error; ER, external rotation; IR, internal rotation; η^2^p, partial eta squared. Notes: Time represents the pre- vs. post-fatigue main effect. The Time × Fatigue Type interaction tests whether the pre-to-post change differs between concentric and eccentric fatigue. Statistical significance was set at *p* < 0.05.

**Table 3:** Kinesthesia (TTDPM): mean ± SD and key ANOVA effects.

Outcome	Concentric Pre	Concentric Post	Eccentric Pre	Eccentric Post	Time	Time × Fatigue Type
TTDPM	5.91 ± 3.64	7.39 ± 6.29	5.18 ± 4.03	6.70 ± 5.82	F(1,77) = 10.86, *p* = 0.001, η^2^p = 0.12	F(1,77) = 0.00, *p* = 0.968, η^2^p = 0.00

Abbreviations: TTDPM, threshold to detection of passive movement; η^2^p, partial eta squared. Notes: Time represents the pre- vs. post-fatigue main effect. The Time × Fatigue Type interaction tests whether the pre-to-post change differs between protocols.

**Table 4:** Y Balance Test–Upper Quarter (YBT-UQ): selected outcomes (mean ± SD) and key ANOVA effects.

Outcome	Concentric Pre	Concentric Post	Eccentric Pre	Eccentric Post	Time	Fatigue Type
Dominant anteromedial	94.54 ± 10.95	92.78 ± 11.05	90.61 ± 8.28	87.76 ± 8.80	F(1,77) = 9.27, *p* = 0.003, η^2^p = 0.11	F(1,77) = 4.26, *p* = 0.042, η^2^p = 0.05
Dominant inferolateral	83.41 ± 12.59	80.93 ± 12.28	84.52 ± 11.55	78.55 ± 10.67	F(1,77) = 15.91, *p* < 0.001, η^2^p = 0.17	F(1,77) = 0.06, *p* = 0.799, η^2^p = 0.00
Dominant superolateral	65.13 ± 10.53	62.20 ± 12.87	64.88 ± 10.56	60.76 ± 12.02	F(1,77) = 24.49, *p* < 0.001, η^2^p = 0.24	F(1,77) = 0.11, *p* = 0.741, η^2^p = 0.00
Dominant composite	87.26 ± 11.37	84.70 ± 11.64	86.18 ± 9.69	81.53 ± 9.93	F(1,77) = 24.07, *p* < 0.001, η^2^p = 0.24	F(1,77) = 0.80, *p* = 0.373, η^2^p = 0.01
Non-dominant anteromedial	92.61 ± 11.13	93.20 ± 10.80	90.12 ± 7.43	89.27 ± 9.64	F(1,77) = 0.00, *p* = 0.986, η^2^p = 0.00	F(1,77) = 2.17, *p* = 0.145, η^2^p = 0.03
Non-dominant superolateral	67.02 ± 11.04	65.59 ± 11.70	64.61 ± 11.40	62.33 ± 11.63	F(1,77) = 6.30, *p* = 0.014, η^2^p = 0.08	F(1,77) = 1.28, *p* = 0.262, η^2^p = 0.02
Non-dominant composite	87.29 ± 10.58	86.01 ± 10.78	85.39 ± 9.93	83.05 ± 9.63	F(1,77) = 5.85, *p* = 0.018, η^2^p = 0.07	F(1,77) = 1.18, *p* = 0.281, η^2^p = 0.02
Non-dominant inferolateral	83.50 ± 9.64	80.76 ± 11.16	83.00 ± 11.25	79.88 ± 10.49	F(1,77) = 9.65, *p* = 0.003, η^2^p = 0.11	F(1,77) = 0.10, *p* = 0.757, η^2^p = 0.00

Abbreviations: YBT-UQ, Y Balance Test–Upper Quarter; dominant, right upper limb; non-dominant, left upper limb; η^2^p, partial eta squared. Notes: Time represents the pre- vs. post-fatigue main effect. Fatigue Type represents the overall difference between concentric and eccentric groups. No Time × Fatigue Type interactions were statistically significant for YBT-UQ outcomes (all *p* > 0.05).

**Table 5:** Athletic Shoulder (ASH) test: isometric force output (mean ± SD) and key ANOVA effects.

Outcome	Concentric Pre	Concentric Post	Eccentric Pre	Eccentric Post	ANOVA Effects
I (Right)	13.02 ± 3.49	11.86 ± 3.33	11.89 ± 3.05	10.52 ± 2.28	Time: F(1,77) = 14.62, *p* < 0.001, η^2^p = 0.16 Time × Type: F(1,77) = 0.11, *p* = 0.745, η^2^p = 0.00
Y (Right)	10.59 ± 2.45	10.10 ± 2.48	10.72 ± 2.15	9.29 ± 2.00	Time: F(1,77) = 18.08, *p* < 0.001, η^2^p = 0.19 Time × Type: F(1,77) = 4.90, *p* = 0.030, η^2^p = 0.06
T (Right)	9.42 ± 2.01	9.05 ± 2.03	9.81 ± 1.62	8.79 ± 1.74	Time: F(1,77) = 16.57, *p* < 0.001, η^2^p = 0.18 Time × Type: F(1,77) = 4.01, *p* = 0.049, η^2^p = 0.05
I (Left)	12.70 ± 3.32	11.27 ± 3.04	11.48 ± 2.37	10.61 ± 2.31	Time: F(1,77) = 43.42, *p* < 0.001, η^2^p = 0.36 Time × Type: F(1,77) = 2.32, *p* = 0.132, η^2^p = 0.03
Y (Left)	9.85 ± 2.27	9.07 ± 2.28	9.90 ± 2.18	9.08 ± 2.36	Time: F(1,77) = 14.63, *p* < 0.001, η^2^p = 0.16 Time × Type: F(1,77) = 0.01, *p* = 0.917, η^2^p = 0.00
T (Left)	9.21 ± 1.97	8.47 ± 2.13	9.04 ± 1.65	8.58 ± 2.39	Time: F(1,77) = 10.10, *p* = 0.002, η^2^p = 0.12 Time × Type: F(1,77) = 0.49, *p* = 0.485, η^2^p = 0.01

Abbreviations: ASH, Athletic Shoulder (ASH) test; I, full abduction; Y, 135° abduction; T, 90° abduction; η^2^p, partial eta squared. Notes: The ANOVA effects column reports the main effect of Time (pre vs. post) and the Time × Fatigue Type interaction (difference in pre-to-post change between protocols). Statistical significance was set at *p* < 0.05.

## Data Availability

The datasets generated and/or analyzed during the current study are available from the corresponding author upon reasonable request. The data are not publicly available due to ethical restrictions and participant privacy considerations.

## Supplementary Materials

The following supporting information can be downloaded at: https://www.mdpi.com/article/10.3390/life16030429/s1, Table S1. Fatigue confirmation metrics and torque-decline patterns for the concentric and eccentric fatigue protocols.

## Abbreviations

AAE	absolute angular error
AM	anteromedial
ANOVA	analysis of variance
ASH	Athletic Shoulder (ASH) test
df	degrees of freedom
EM means	estimated marginal means
ER	external rotation
Hz	Hertz
ICC	intraclass correlation coefficient
IL	inferolateral
IR	internal rotation
JRS	joint repositioning sense
SD	standard deviation
SL	superolateral
TTDPM	threshold to detection of passive movement
YBT-UQ	Y Balance Test–Upper Quarter
η^2^p	partial eta squared
I	“I” position (full abduction)
Y	“Y” position (135° abduction)
T	“T” position (90° abduction)
jamovi	jamovi statistical software
GPower	GPower statistical power analysis software

## Appendix A. Statistical Verification from Raw Data

Table A1, Table A2 and Table A3 provide full mixed-design ANOVA outputs (Time, Fatigue Type, and Time × Fatigue Type) re-computed from the shared dataset (*N* = 79; df_error = 77).

**Table A1:** Joint repositioning sense (JRS) and kinesthesia (TTDPM): full ANOVA outputs.

Outcome	ANOVA Outputs
ER15° AAE	Time: F = 114.49, *p* < 0.001, η^2^p = 0.60 Fatigue Type: F = 1.42, *p* = 0.236, η^2^p = 0.02 Time × Type: F = 1.95, *p* = 0.166, η^2^p = 0.02
ER30° AAE	Time: F = 105.37, *p* < 0.001, η^2^p = 0.58 Fatigue Type: F = 0.08, *p* = 0.776, η^2^p = 0.00 Time × Type: F = 0.02, *p* = 0.890, η^2^p = 0.00
ER45° AAE	Time: F = 68.74, *p* < 0.001, η^2^p = 0.47 Fatigue Type: F = 3.13, *p* = 0.081, η^2^p = 0.04 Time × Type: F = 2.16, *p* = 0.146, η^2^p = 0.03
IR15° AAE	Time: F = 38.01, *p* < 0.001, η^2^p = 0.33 Fatigue Type: F = 1.34, *p* = 0.251, η^2^p = 0.02 Time × Type: F = 0.01, *p* = 0.924, η^2^p = 0.00
IR30° AAE	Time: F = 40.72, *p* < 0.001, η^2^p = 0.35 Fatigue Type: F = 0.01, *p* = 0.929, η^2^p = 0.00 Time × Type: F = 3.79, *p* = 0.055, η^2^p = 0.05
IR45° AAE	Time: F = 14.03, *p* < 0.001, η^2^p = 0.15 Fatigue Type: F = 0.04, *p* = 0.849, η^2^p = 0.00 Time × Type: F = 5.91, *p* = 0.017, η^2^p = 0.07
KinER-to-IR TTDPM	Time: F = 10.86, *p* = 0.001, η^2^p = 0.12 Fatigue Type: F = 0.45, *p* = 0.506, η^2^p = 0.01 Time × Type: F = 0.00, *p* = 0.968, η^2^p = 0.00

**Table A2:** Y Balance Test–Upper Quarter (YBT-UQ): full ANOVA outputs.

Outcome	ANOVA Outputs
YBT-UQ Anteromedial (Dom)	Time: F = 9.27, *p* = 0.003, η^2^p = 0.11 Fatigue Type: F = 4.26, *p* = 0.042, η^2^p = 0.05 Time × Type: F = 0.54, *p* = 0.463, η^2^p = 0.01
YBT-UQ Inferolateral (Dom)	Time: F = 15.91, *p* < 0.001, η^2^p = 0.17 Fatigue Type: F = 0.06, *p* = 0.799, η^2^p = 0.00 Time × Type: F = 3.04, *p* = 0.085, η^2^p = 0.04
YBT-UQ Superolateral (Dom)	Time: F = 24.49, *p* < 0.001, η^2^p = 0.24 Fatigue Type: F = 0.11, *p* = 0.741, η^2^p = 0.00 Time × Type: F = 0.71, *p* = 0.401, η^2^p = 0.01
YBT-UQ Composite (Dom)	Time: F = 24.07, *p* < 0.001, η^2^p = 0.24 Fatigue Type: F = 0.80, *p* = 0.373, η^2^p = 0.01 Time × Type: F = 2.17, *p* = 0.145, η^2^p = 0.03
YBT-UQ Anteromedial (Non-dom)	Time: F = 0.00, *p* = 0.986, η^2^p = 0.00 Fatigue Type: F = 2.17, *p* = 0.145, η^2^p = 0.03 Time × Type: F = 0.99, *p* = 0.323, η^2^p = 0.01
YBT-UQ Inferolateral (Non-dom)	Time: F = 9.65, *p* = 0.003, η^2^p = 0.11 Fatigue Type: F = 0.10, *p* = 0.757, η^2^p = 0.00 Time × Type: F = 0.04, *p* = 0.841, η^2^p = 0.00
YBT-UQ Superolateral (Non-dom)	Time: F = 6.30, *p* = 0.014, η^2^p = 0.08 Fatigue Type: F = 1.28, *p* = 0.262, η^2^p = 0.02 Time × Type: F = 0.34, *p* = 0.563, η^2^p = 0.00
YBT-UQ Composite (Non-dom)	Time: F = 5.85, *p* = 0.018, η^2^p = 0.07 Fatigue Type: F = 1.18, *p* = 0.281, η^2^p = 0.02 Time × Type: F = 0.53, *p* = 0.468, η^2^p = 0.01

**Table A3:** Athletic Shoulder (ASH) test: full ANOVA outputs.

Outcome	ANOVA Outputs
ASH I (Dominant/Right)	Time: F = 14.62, *p* < 0.001, η^2^p = 0.16 Fatigue Type: F = 3.81, *p* = 0.054, η^2^p = 0.05 Time × Type: F = 0.11, *p* = 0.745, η^2^p = 0.00
ASH Y (Dominant/Right)	Time: F = 18.08, *p* < 0.001, η^2^p = 0.19 Fatigue Type: F = 0.50, *p* = 0.480, η^2^p = 0.01 Time × Type: F = 4.90, *p* = 0.030, η^2^p = 0.06
ASH T (Dominant/Right)	Time: F = 16.57, *p* < 0.001, η^2^p = 0.18 Fatigue Type: F = 0.03, *p* = 0.871, η^2^p = 0.00 Time × Type: F = 4.01, *p* = 0.049, η^2^p = 0.05
ASH I (Non-dominant/Left)	Time: F = 43.42, *p* < 0.001, η^2^p = 0.36 Fatigue Type: F = 2.24, *p* = 0.139, η^2^p = 0.03 Time × Type: F = 2.32, *p* = 0.132, η^2^p = 0.03
ASH Y (Non-dominant/Left)	Time: F = 14.63, *p* < 0.001, η^2^p = 0.16 Fatigue Type: F = 0.00, *p* = 0.948, η^2^p = 0.00 Time × Type: F = 0.01, *p* = 0.917, η^2^p = 0.00
ASH T (Non-dominant/Left)	Time: F = 10.10, *p* = 0.002, η^2^p = 0.12 Fatigue Type: F = 0.01, *p* = 0.936, η^2^p = 0.00 Time × Type: F = 0.49, *p* = 0.485, η^2^p = 0.01

## References

[1] Møller M., Nielsen R.O., Attermann J., Wedderkopp N., Lind M., Sørensen H., Myklebust G. (2017). Handball load and shoulder injury rate: A 31-week cohort study of 679 elite youth handball players. Br. J. Sports Med..

[2] Hadjisavvas S., Efstathiou M.A., Malliou V., Giannaki C.D., Stefanakis M. (2022). Risk factors for shoulder injuries in handball: Systematic review. BMC Sports Sci. Med. Rehabil..

[3] Enoka R.M., Duchateau J. (2016). Translating Fatigue to Human Performance. Med. Sci. Sports Exerc..

[4] Gandevia S.C. (2001). Spinal and supraspinal factors in human muscle fatigue. Physiol. Rev..

[5] LaStayo P.C., Woolf J.M., Lewek M.D., Snyder-Mackler L., Reich T., Lindstedt S.L. (2003). Eccentric muscle contractions: Their contribution to injury, prevention, rehabilitation, and sport. J. Orthop. Sports Phys. Ther..

[6] Skejø S.D., Møller M., Bencke J., Sørensen H. (2019). Shoulder kinematics and kinetics of team handball throwing: A scoping review. Hum. Mov. Sci..

[7] Wagner H., Pfusterschmied J., Von Duvillard S.P., Müller E. (2011). Performance and kinematics of various throwing techniques in team-handball. J. Sports Sci. Med..

[8] Riemann B.L., Lephart S.M. (2002). The Sensorimotor System, Part II: The Role of Proprioception in Motor Control and Functional Joint Stability. J. Athl. Train..

[9] Proske U., Gandevia S.C. (2012). The proprioceptive senses: Their roles in signaling body shape, body position and movement, and muscle force. Physiol. Rev..

[10] Lee H.-M., Liau J.-J., Cheng C.-K., Tan C.-M., Shih J.-T. (2003). Evaluation of shoulder proprioception following muscle fatigue. Clin. Biomech..

[11] Brockett C., Warren N., Gregory J., Morgan D., Proske U. (1997). A comparison of the effects of concentric versus eccentric exercise on force and position sense at the human elbow joint. Brain Res..

[12] Gorman P.P., Butler R.J., Plisky P.J., Kiesel K.B. (2012). Upper Quarter Y Balance Test: Reliability and performance comparison between genders in active adults. J. Strength Cond. Res..

[13] Ribeiro F., Mota J., Oliveira J. (2007). Effect of exercise-induced fatigue on position sense of the knee in the elderly. Eur. J. Appl. Physiol..

[14] Schwiertz G., Brueckner D., Schedler S., Kiss R., Muehlbauer T. (2019). Reliability and Minimal Detectable Change of the Upper Quarter Y-Balance Test in Healthy Adolescents Aged 12 to 17 Years. Int. J. Sports Phys. Ther..

[15] Ashworth B., Hogben P., Singh N., Tulloch L., Cohen D.D. (2018). The Athletic Shoulder (ASH) test: Reliability of a novel upper body isometric strength test in elite rugby players. BMJ Open Sport Exerc. Med..

[16] Allen T.J., Leung M., Proske U. (2010). The effect of fatigue from exercise on human limb position sense. J. Physiol..

[17] Carpenter J.E., Blasier R.B., Pellizzon G.G. (1998). The effects of muscle fatigue on shoulder joint position sense. Am. J. Sports Med..

[18] Taylor J.L., Amann M., Duchateau J., Meeusen R., Rice C.L. (2016). Neural Contributions to Muscle Fatigue: From the Brain to the Muscle and Back Again. Med. Sci. Sports Exerc..

[19] Sidhu S.K., Weavil J.C., Thurston T.S., Rosenberger D., Jessop J.E., Wang E., Richardson R.S., McNeil C.J., Amann M. (2018). Fatigue-related group III/IV muscle afferent feedback facilitates intracortical inhibition during locomotor exercise. J. Physiol..

[20] Plisky P.J., Gorman P.P., Butler R.J., Kiesel K.B., Underwood F.B., Elkins B. (2009). The reliability of an instrumented device for measuring components of the star excursion balance test. N. Am. J. Sports Phys. Ther..

[21] Andersson S.H., Bahr R., Clarsen B., Myklebust G. (2017). Preventing overuse shoulder injuries among throwing athletes: A cluster-randomised controlled trial in 660 elite handball players. Br. J. Sports Med..

[22] Asker M., Hägglund M., Waldén M., Källberg H., Skillgate E. (2022). The Effect of Shoulder and Knee Exercise Programmes on the Risk of Shoulder and Knee Injuries in Adolescent Elite Handball Players: A Three-Armed Cluster Randomised Controlled Trial. Sports Med.—Open.

[23] Fleisig G.S., Bolt B., Fortenbaugh D., Wilk K.E., Andrews J.R. (2011). Biomechanical comparison of baseball pitching and long-toss: Implications for training and rehabilitation. J. Orthop. Sports Phys. Ther..

