# Supplementary material for: Concentric Versus Eccentric Exercise-Induced Fatigue on Proprioception, Motor Control and Performance of the Upper Limb in Handball Players: A Retrospective Study
Source: Life (Basel). 2026 Mar 6;16(3):429. doi: 10.3390/life16030429 (PMC13027890; doi:10.3390/life16030429)
Supplement: Supplementary file 1 [file life-16-00429-s001.zip › life-4130636-supplementary.pdf]

Table S1. Fatigue confirmation metrics and torque-decline patterns for the concentric and eccentric fatigue protocols.

Protocol	n	Movement	Metric	Value (mean ± SD [range])
Concentric	46	IR	First 10 reps	35.48 ± 7.32 (21.55–54.25)
Concentric	46	IR	Last 10 reps	13.53 ± 5.16 (2.12–30.28)
Concentric	46	IR	% drop (First10→Last10)	62.05 ± 11.82 (28.57–90.15)
Concentric	46	ER	First 10 reps	22.75 ± 5.65 (11.65–33.35)
Concentric	46	ER	Last 10 reps	8.90 ± 3.27 (1.71–15.90)
Concentric	46	ER	% drop (First10→Last10)	60.00 ± 14.86 (12.54–87.21)
Eccentric	33	IR	Before (reference)	48.52 ± 10.46 (33.40–71.20)
Eccentric	33	IR	After (post-fatigue)	16.24 ± 3.38 (11.10–25.48)
Eccentric	33	IR	% drop (Before→After)	65.79 ± 6.64 (51.63–81.09)
Eccentric	33	ER	Before (reference)	32.50 ± 8.02 (19.35–52.70)
Eccentric	33	ER	After (post-fatigue)	10.12 ± 2.76 (7.37–17.96)
Eccentric	33	ER	% drop (Before→After)	67.93 ± 8.25 (42.23–83.19)
